# Immediate lymphatic reconstruction for the prevention of breast cancer-related lymphedema: an experience highlighting the importance of lymphatic anatomy

**DOI:** 10.20517/2347-9264.2022.100

**Published:** 2023-05-23

**Authors:** Rosie Friedman, Jacquelyn R. Kinney, Aneesh Bahadur, Dhruv Singhal

**Affiliations:** Division of Plastic and Reconstructive Surgery, Department of Surgery, Beth Israel Deaconess Medical Center, Harvard Medical School, Boston, MA 02215, USA.

**Keywords:** Lymphatic anatomy, immediate lymphatic reconstruction, lymphedema

## Abstract

Immediate lymphatic reconstruction (ILR) has become increasingly utilized for the prevention of breast cancer-related lymphedema (BCRL). A growing body of evidence has demonstrated the long-term efficacy of ILR in reducing the rate of BCRL. While certain risk factors for BCRL are well-recognized, such as axillary lymph node dissection, regional lymph node radiation, and elevated body mass index, other potential risk factors such as age and taxane-based chemotherapeutics remain under discussion. Our experience with ILR has highlighted an additional potential risk factor for BCRL. Lymphatic anatomy, specifically compensatory lymphatic channels that bypass the axilla, may play a largely underrecognized role in determining which patients will develop BCRL after ILR. Foundational anatomic knowledge has primarily been based on cadaveric studies that predate the twentieth century. Modern approaches to lymphatic anatomical mapping using indocyanine green lymphography have helped to elucidate baseline lymphatic anatomy and compensatory channels, and certain variations within these channels may act as anatomic risk factors. Therefore, the purpose of this review was to highlight ways in which variations in lymphatic anatomy can inform the application and improve the accessibility of this procedure. As ILR continues to advance and evolve, anatomical mapping of the lymphatic system is valuable to both the patient and lymphatic microsurgeon and is a critical area of future study.

## INTRODUCTION

A significant survivorship issue following breast cancer treatment is breast cancer-related lymphedema (BCRL). BCRL arises due to the accumulation of lymphatic fluid in the upper extremity as a result of damage to the lymphatic system during axillary lymph node dissection (ALND)^[1]^. The fluid accumulation can result in disfiguring edema, erythema, pain, tightness, heaviness, and diminished function of the affected extremity^[2,3]^. If left untreated, BCRL is typically progressive and can be complicated by life-threatening infections. In addition to distressing physical symptoms, patients may face psychosocial burdens secondary to BCRL^[4,5,6]^. Additionally, patients with BCRL face considerable out-of-pocket costs irrespective of treatment modality^[7,8]^.

The incidence of BCRL following axillary lymph node dissection is reported to be between 21% to 34%^[9–14]^. Variation in reported incidence may be due to the lack of standardization in methods of assessment and diagnostic criteria. Notably, the incidence of lymphedema is disproportionately higher among Black and Hispanic patient populations, highlighting a healthcare disparity among breast cancer survivors^[15]^. Breast cancer mortality rates have declined due to advancements in diagnostic modalities and clinical management^[16]^. Therefore, the rates of BCRL can be expected to increase in the coming decades and there remains an unmet need for physicians and researchers dedicated to the prevention and treatment of this disease^[17]^.

The pathophysiology of BCRL occurs through three stages: fluid accumulation, fibrosis, and fatty tissue deposition. In the initial stages, interstitial fluid stasis takes place and proliferation of inflammatory cells ensues^[18]^. This inflammatory response leads to lymphatic vessel deterioration, fibrosis, and inhibition of lymphangiogenesis^[19–22]^. Lastly, subcutaneous adipose tissue is deposited^[23,24]^. Notably, multiple genes have been implicated in the development of BCRL, including *HGF* and *GJC2* genes^[25–28]^. This knowledge has been utilized clinically by recommending genetic testing for patients for earlier detection of lymphedema, though further research is warranted^[29]^.

As the underlying inciting event of BCRL development is the disruption of lymphatic vessels during oncologic surgery, our team has focused on the operative prevention of BCRL. The purpose of this review is to highlight ways in which variations in lymphatic anatomy can inform the application and improve the accessibility of the surgical prevention of lymphedema. In order to adequately discuss surgical prevention, it is important to first understand identifiable preoperative risk factors.

## RISK FACTORS FOR DEVELOPING BCRL

The single greatest risk factor is ALND. Patients who undergo ALND are at a substantially higher risk of developing BCRL, with a relative risk of 3.47 in comparison to those who do not require ALND for oncologic treatment^[11,30,31]^. Findings from Yusof *et al.* determined that ten or more excised lymph nodes was associated with a three-fold increased risk of BCRL, due to more extensive damage to the lymphatic vessels^[32]^. Furthermore, patients with a larger burden of oncologic disease within the lymph nodes may be at higher risk of BCRL development, as the invasion of cancer cells within the lymph nodes may overcrowd and disrupt normal lymphatic architecture, thereby impairing lymphatic flow^[30,33]^.

Regional lymph node radiation (RLNR) substantially increases a patient’s risk of BCRL in a delayed manner, as it can take months or years for radiation-related fibrosis to develop^[34,35]^. The development of fibrosis within the lymph node can compress and distort the lymphatic tissue, resulting in increased fluid accumulation in the distal lymphatics^[36,37]^. RLNR targeted at supraclavicular or axillary lymph nodes presents the greatest risk of BCRL, whereas the risk after chest wall radiotherapy appears to be lower^[37]^.

Body mass index (BMI) is recognized as the primary modifiable risk factor linked to the development of BCRL^[30,38–40]^. A higher BMI has been positively correlated with the development of BCRL, with obese patients having a greater risk of developing lymphedema compared to those who are overweight or within the normal range^[39]^. This correlation may be explained by underlying biochemical changes to the lymphatic system in patients with higher BMI, including inflammatory processes and direct injury to lymphatic endothelial cells, which likely induce baseline lymphatic disruption^[41]^.

There are other important risk factors that remain controversial. Multiple studies have reported an association between taxane-based chemotherapeutic administration and BCRL development^[42–46]^, while other studies have not supported this finding^[47]^. Cariati *et al.* demonstrated that the use of adjuvant taxane-based chemotherapy conferred a threefold increase in the risk of BCRL development^[45]^. In a large prospective study, Swaroop *et al.* noted that adjuvant docetaxol increased the risk of mild swelling though taxane-based chemotherapy was not a risk factor for BCRL development^[47]^. Fewer studies have focused on examining the effects of neoadjuvant taxane-based chemotherapy on the development of BCRL^[48–50]^. Johnson *et al.* demonstrated that patients who received neoadjuvant taxane-based chemotherapy had a reduction in lymphatic contractile function and demonstrated a possible association with the presence of peripheral neuropathy in those who received neoadjuvant taxane-based chemotherapy^[48]^.

Multiple prior studies have noted an association between increasing age and BCRL^[51–53]^. Shang *et al.* demonstrated that aging results in loss of muscle cells, impairment of lymphatic contractile function, and increased production of inflammatory cytokines^[54]^. However, other studies offer contradictory findings, with some reporting that the incidence of BCRL is higher in younger women^[55–57]^.

There is uncertainty as to how factors pertaining to oncologic breast surgery, such as the extent of breast surgery and reconstruction, may modify individual risk of BCRL. A previous investigation reported that modified radical mastectomy appeared to be an independent risk factor for BCRL^[58]^. Other studies have indicated that the rate of BCRL was higher in those who underwent a total mastectomy compared to those who underwent partial mastectomy^[59]^. Additionally, patients undergoing multiple surgeries including both mastectomy and lumpectomy on the same breast are likely at higher risk of BCRL than those having only one procedure alone^[32]^. In addition, multiple studies have examined the relationship between breast reconstruction and BCRL development. In a meta-analysis, Siotos *et al.* determined that breast reconstruction was associated with a lower risk of lymphedema compared to mastectomy alone^[60]^. In a matched cohort study of over 400 patients, Basta *et al.* reported that immediate breast reconstruction did seem to influence the risk of BCRL development^[61]^. Though the influence of breast reconstruction on the risk of BCRL development is not fully understood, breast reconstruction does not appear to adversely affect the risk of BCRL^[62]^.

## IMMEDIATE LYMPHATIC RECONSTRUCTION

Lymphovenous bypass (LVB), as described by Yukio Yamada in 1969 as a surgical treatment for chronic lymphedema, was the first successful surgical technique developed to restore lymphatic flow in an animal model^[63]^. In this study, a successful anastomosis of the thoracic duct into the venous system was created, thereby restoring a novel afferent route for lymphatic fluid. In 2009, Boccardo *et al.* applied this innovation for the prevention of BCRL by rerouting arm lymphatics to an axillary vein tributary at the time of ALND^[64]^. This procedure, originally termed Lymphatic Microsurgical Preventative Healing Approach (LYMPHA)^[64,65]^, has more recently been referred to as immediate lymphatic reconstruction^[66]^. A growing body of evidence has demonstrated promising results of ILR for the prevention of BCRL, including a recent meta-analysis which reported a BCRL incidence of 5.7% in patients who underwent ILR compared to 34% in those who underwent ALND alone^[13]^.

**Figure 1.**
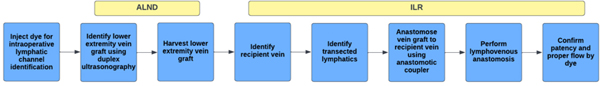
Comprehensive workflow of immediate lymphatic reconstruction (ILR) following axillary lymph node dissection (ALND).

An overview of the steps of immediate lymphatic reconstruction is outlined in Figure 1. Immediately prior to ALND, a lymphatic-specific dye is injected intradermally for lymphatic channel identification. In order to ensure comprehensive visualization of lymphatic channels, we perform intradermal injections of 0.25cc of 2% fluorescein isothiocyanate (FITC) mixed with albumin at the 1^st^ and 4^th^ dorsal hand web spaces and at the radial and ulnar aspects of the volar wrist crease. Additionally, 1cc of isosulfan blue is injected intradermally over the course of the cephalic vein, identified by ultrasound, in the lateral upper arm. The anatomic location of these injections can vary, with some opting for upper arm injections as originally described^[67,68]^. Once the ALND is complete, the dye allows for visualization of disrupted lymphatic channels within the axilla and the channels can then be prepared for the LVB.

Various dyes have been utilized for identification of lymphatic channels, including isosulfan blue, indocyanine green (ICG), and FITC^[67]^. Isosulfan blue dye was initially used for ILR, but this dye is also frequently utilized for the oncologic mapping of sentinel lymph nodes; therefore, this presented challenges in distinguishing sentinel lymph nodes from peripheral arm lymphatics. This necessitated the adoption of novel dyes for lymphatic channel identification, such as ICG, which remains a favorable option as it is not consistently used during the oncologic portion of the procedure. However, the use of ICG is limited by the inability to visualize the dye without a near-infrared camera and can compromise the surgeon’s view of the surrounding structures under the surgical microscope. Additionally, some oncologic surgeons will utilize ICG for breast sentinel lymph node biopsy, though this is institution dependent. Some groups have utilized FITC as an effective alternative, given the ability of FITC to be visualized with a fluorescence filter applied to the microscope that does not limit the visibility of surrounding anatomical structures^[69]^. Therefore, both lymphatic channel visualization and microsurgical reconstruction can be carried out without interference. Notably, each of these techniques allows for visualization of superficial structures 1–2 cm below the skin and therefore, deep lymphatic channels are not currently able to be readily identified during ILR.

While each dye has distinct advantages and disadvantages, further research is necessary to develop standardized methods for lymphatic channel identification^[70]^. For example, increasing dye uptake in lymphatic vessels and improved visualization of deep lymphatic channels are notable obstacles in the application of newer dyes. Conjugating a fluorophore to a larger compound, such as to dextran, albumin, or polyethylene glycol (PEG), may have potential utilization, as any particle too small (< 5 nm) or too large (> 100 nm) precludes dye uptake into the lymphatic channels^[71]^. Prior investigations determined that the optimal size for lymphatic uptake is 10–100 nm; therefore, these dyes may aid in optimizing lymphatic uptake. Furthermore, near-infrared (NIR) dyes and upconverting nanoparticles (UCNPs) are other potential methods to enhance lymphatic visualization^[72]^.

**Figure 2.**
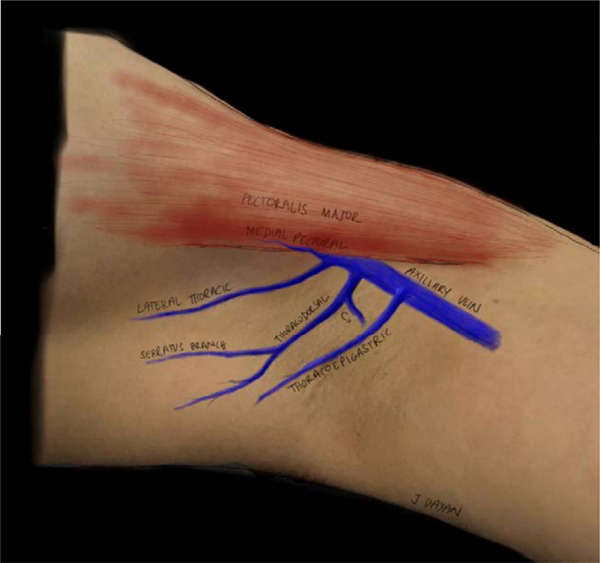
Potential recipient vein options in the axilla for immediate lymphatic reconstruction (reused with permission, Coriddi *et al.*, 2020, Plastic and Reconstructive Surgery Global Open^[67]^).

Following the identification of the transected lymphatic channels, a target vein for the lymphovenous bypass is identified. There are multiple recipient venous candidates in the axilla, including the accessory vein (thoracoepigastric vein), lateral thoracic vein, medial pectoral vein, circumflex scapular vein, thoracodorsal vein, or other unnamed adjacent venous tributaries [Figure 2]^[67,73]^. The accessory vein, which is the most popular for ILR, is found coursing through the level 1 axillary lymph nodes, originating perpendicular from the axillary vein, 2 cm anterior to the thoracodorsal vessels. Due to its proximity to arm lymphatic channels, it has become an ideal candidate for the procedure^[67]^. Unfortunately, this proximity to the axillary lymph nodes also places this vein at risk for transection and removal during axillary lymph node excision. In this case, any of the previously mentioned veins can be used as an alternative^[74,75]^.

The recipient vein requires adequate length, which we have found to be ideally ≥ 5cm, as it must be long enough to reach the arm lymphatic vessels while avoiding undue tension on the anastomosis. The presence of at least one venous valve is vital for preventing venous back-bleeding through the site of the anastomosis. Significant back-bleeding can overwhelm the lymphatic system, given the pressure differential across the anastomosis, thereby preventing afferent lymphatic flow. Furthermore, the size of the recipient vein is a critical consideration as the lymphatic channels are significantly smaller than that of their venous counterparts. To help alleviate this size discrepancy, multiple lymphatic channels can be intussuscepted into the vein, or if the lymphatic vessels are large enough, an end-to-end anastomosis can be performed with a small vein^[67,76]^. Utilization of venous branches of the recipient vein has also become an effective method to optimize the size-matching of the lymphatic channel to the recipient vein^[67]^. Moreover, each branch point is likely to contain a valve, thereby further preventing the backflow of venous blood^[77]^. Of note, unlike lymphovenous bypasses for chronic lymphedema performed in the distal extremity where preoperative ultrasound can assist in identifying reflux-free veins^[74,75]^, this is not possible pre-operatively in preventative cases as the veins are deeper and their availability and physiology may be altered following lymphadenectomy. Even with careful consideration and selection of the recipient vein, venous back-bleeding and inadequate recipient vein length are two technical challenges that impede the success of ILR and lead to aborting procedures intraoperatively. Recently, our team has instituted routine use of a lower extremity vein graft to overcome these venous-related complications^[78]^. In this technique, a 5 cm target vein is identified by ultrasound as a superficial secondary or tertiary branch of the greater saphenous vein in the medial lower leg, caudal to the medial epicondyle of the knee. This segment is ideally selected to ensure the presence of at least two branches or one venous valve, which can be visualized on ultrasonography. The vein is then harvested and anastomosed to the axillary vein tributary, maintaining the orientation of the vein graft in order to preserve the proper directionality of the venous valve. Since utilizing a lower extremity vein graft during ILR, our intraoperative aborted case rate was reduced from 14% to 0%, thereby suggesting the promising effects and potential utility of this innovation to mitigate venous-related complications^[78]^. Furthermore, the harvest of the lower extremity vein graft was performed synchronously with the ALND and therefore did not increase the intraoperative time of the overall operation^[78]^.

Of note, additional preventative surgical approaches to reducing the risk of lymphedema have been proposed, including peripheral supermicrosurgical anastomoses and prophylactic lymph node transplantations and lymphatic flaps^[79–83]^. Prophylactic peripheral lymphovenous bypasses offer an interesting approach which would essentially eliminate the effect of adjuvant radiotherapy which is usually targeted to the nodal region. The challenge of this prophylactic approach is identifying anatomically which lymphatic channels should be bypassed. Prophylactic lymph node transplantations and lymphatic flaps offer a promising approach. However, the surgeon must carefully balance the morbidity of the donor site with the relative risk reduction of lymphedema development^[83,84]^.

## LYMPHATIC ANATOMY

Despite continued evidence demonstrating the effectiveness of ILR for the prevention of BCRL, there are several barriers that may hinder the progress and advancement of this approach within the field of lymphatic surgery. Firstly, ILR remains a technically demanding procedure that is not frequently covered by health insurance^[85]^. Additionally, there are a limited number of lymphatic centers and surgeons formally trained in lymphatic microsurgery, and therefore patients are often required to travel long distances to undergo ILR^[86]^. While the incidence of BCRL after ALND and RLND approaches 25–30%, around 70% of patients do not ever develop lymphedema. Although the occurrence of BRCL may be moderate, counseling all patients regarding the risk of lymphedema after oncologic surgery is necessary for proper patient management. In addition, discussing the benefits of ILR and obtaining thorough informed consent enhances patient autonomy and understanding of medical information^[87]^. Importantly, identifying the individuals with the highest risk for BCRL development will allow us to overcome resource constraints and deliver this procedure to those who need it the most.

**Figure 3.**
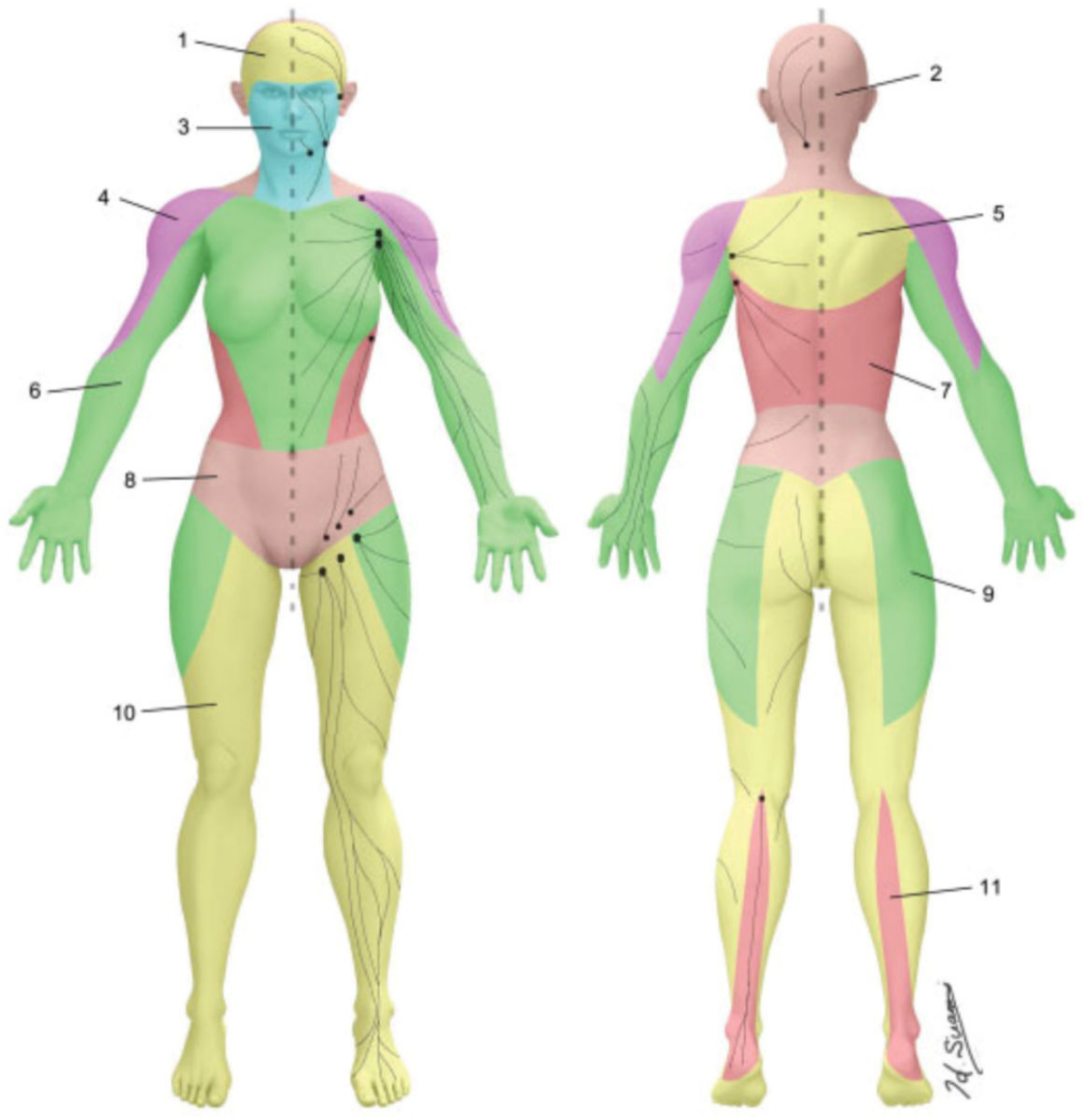
Major lymphosomes of the body (reused with permission, Suami *et al.* 2018, Seminars in Plastic Surgery^[90]^).

We believe that a better understanding of individual variations in lymphatic anatomy will help identify those patients in greatest need for ILR. To date, there is no modern comprehensive compendium or map of normal lymphatic anatomy and most of our current foundational knowledge has been obtained from cadaveric dissections that predate the twentieth century^[88]^. However, more recent efforts have been made to further the anatomic knowledge of the lymphatic system. In 2016, Suami *et al.* described the lymphosome concept [Figure 3], which is defined as predictable areas of the body in which the lymphatics will reliably drain to a designated group of lymph nodes^[89,90]^. This concept has advanced our understanding of lymphatic anatomy and allowed for more accurate predictions regarding the location of major lymphatic channels. A detailed appreciation of lymphatic anatomy based on the lymphosome concept may help guide lymphatic surgeons in selecting which lymphatic channels to bypass when multiple transected channels are identified intraoperatively and knowledge of lymphatic anatomy in relation to venous vasculature may facilitate lymphovenous bypass^[91]^.

Based on delineated lymphosomes, in our experience with ILR, we have noted that different regions of the upper extremity drain to distinct areas of the axilla. We previously investigated lymphosomes of the upper extremity using two distinct dyes, FITC and isosulfan blue, in order to differentiate medial and lateral upper arm lymphosomes^[92]^. In this study, we demonstrated that the lateral upper arm drained via a lymphatic channel that did not course through the axilla in the vast majority of patients^[92]^. This pathway was distinct from those of the medial upper arm, which reliably were identified as draining to the axilla. Given its extraaxillary drainage, the lateral upper arm channel had previously been described as one of the few compensatory routes of lymphatic drainage following ALND, which was further supported by our study^[92]^. The lateral upper arm channel, along with other compensatory drainage routes that bypass the axilla, are postulated to be protective against BCRL and may help to explain why only a percentage of patients undergoing the same oncologic treatments ultimately go on to develop BCRL. This finding has focused our group on lymphatic anatomy as we believe characterization of baseline anatomy and compensatory channels will help to predict which patients will develop BCRL after ALND.

A surgical prevention program cannot exist without a comprehensive surveillance protocol involving a multidisciplinary preoperative assessment. As part of our program’s preoperative assessment, we routinely perform ICG lymphography prior to ALND and ILR in order to visualize and map baseline superficial lymphatic anatomy. Over time, our group became increasingly focused on the visualization of compensatory lymphatic channels on ICG and this informed our ICG injection sites such that we implemented targeted ICG injection sites to capture these channels^[93]^. Early in our ICG experience, we performed two anterior ICG injections in the wrist crease and two posterior injections at the first and fourth webspace of the hand. However, we later refined our injection technique to include an additional injection over the cephalic vein, which allowed us to reliably visualize the lateral upper arm channel^[94]^. Additionally, we have more recently added a peri-olecranon injection to visualize another compensatory channel: the tricipital or Caplan’s pathway^[95–99]^.

**Figure 4.**
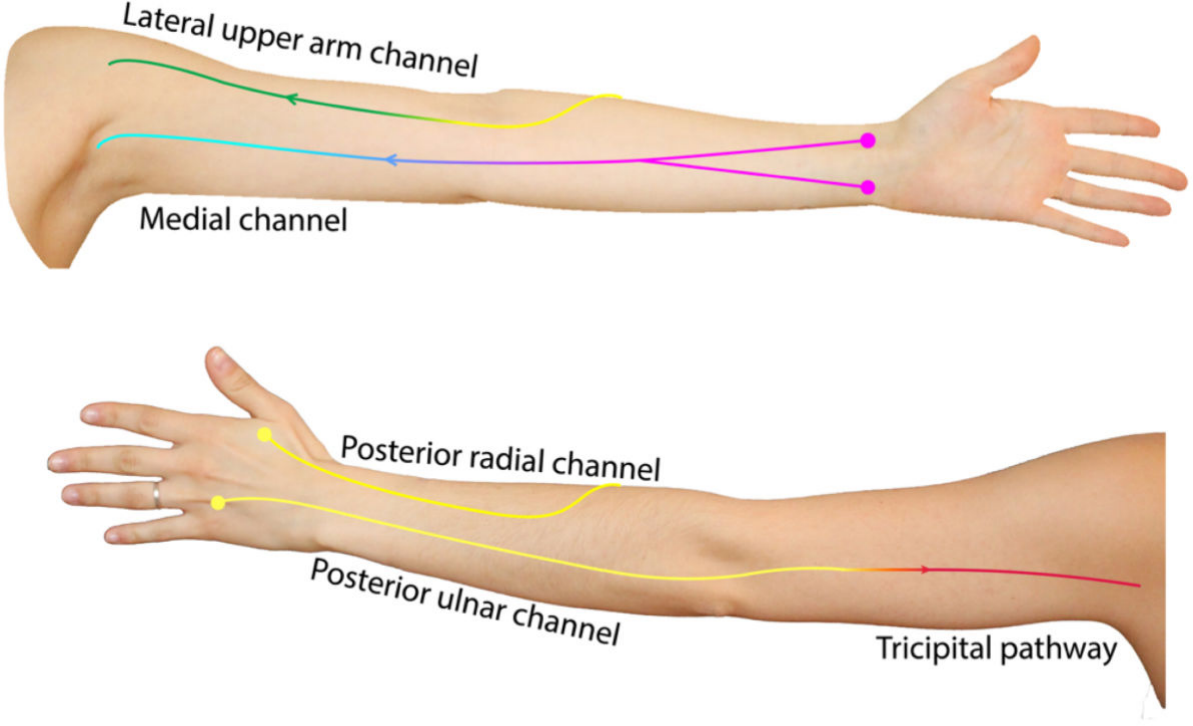
Schematic demonstrating the most frequently observed baseline lymphatic anatomy as visualized on preoperative ICG lymphography; colored circles represent webspace and wrist crease ICG injection sites.

**Figure 5.**
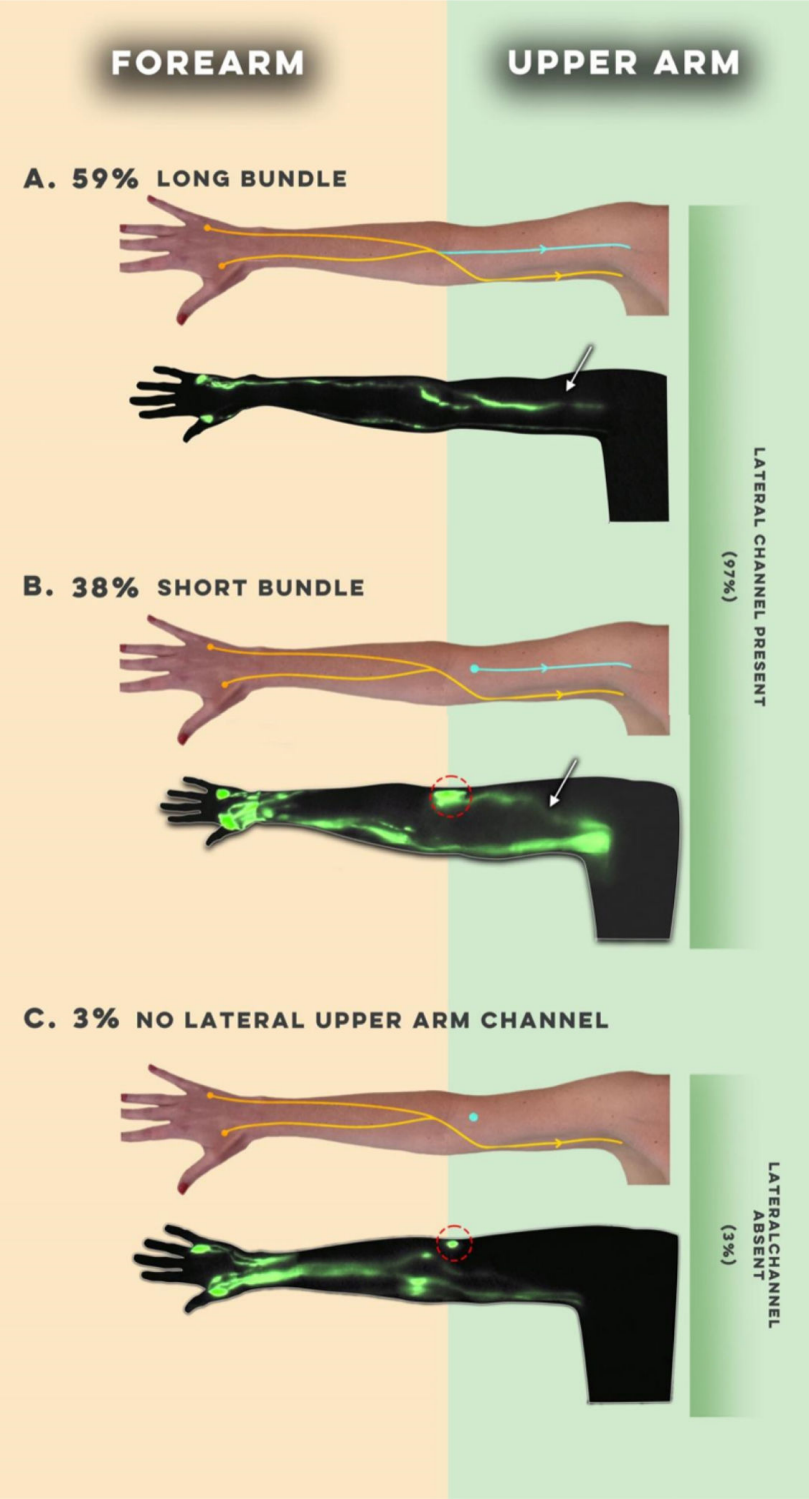
Long and short bundle phenotypes of the lateral upper arm lymphatic channel (reused with permission, Granoff *et al.* 2022, Plastic and Reconstructive Surgery^[101]^).

**Figure 6.**
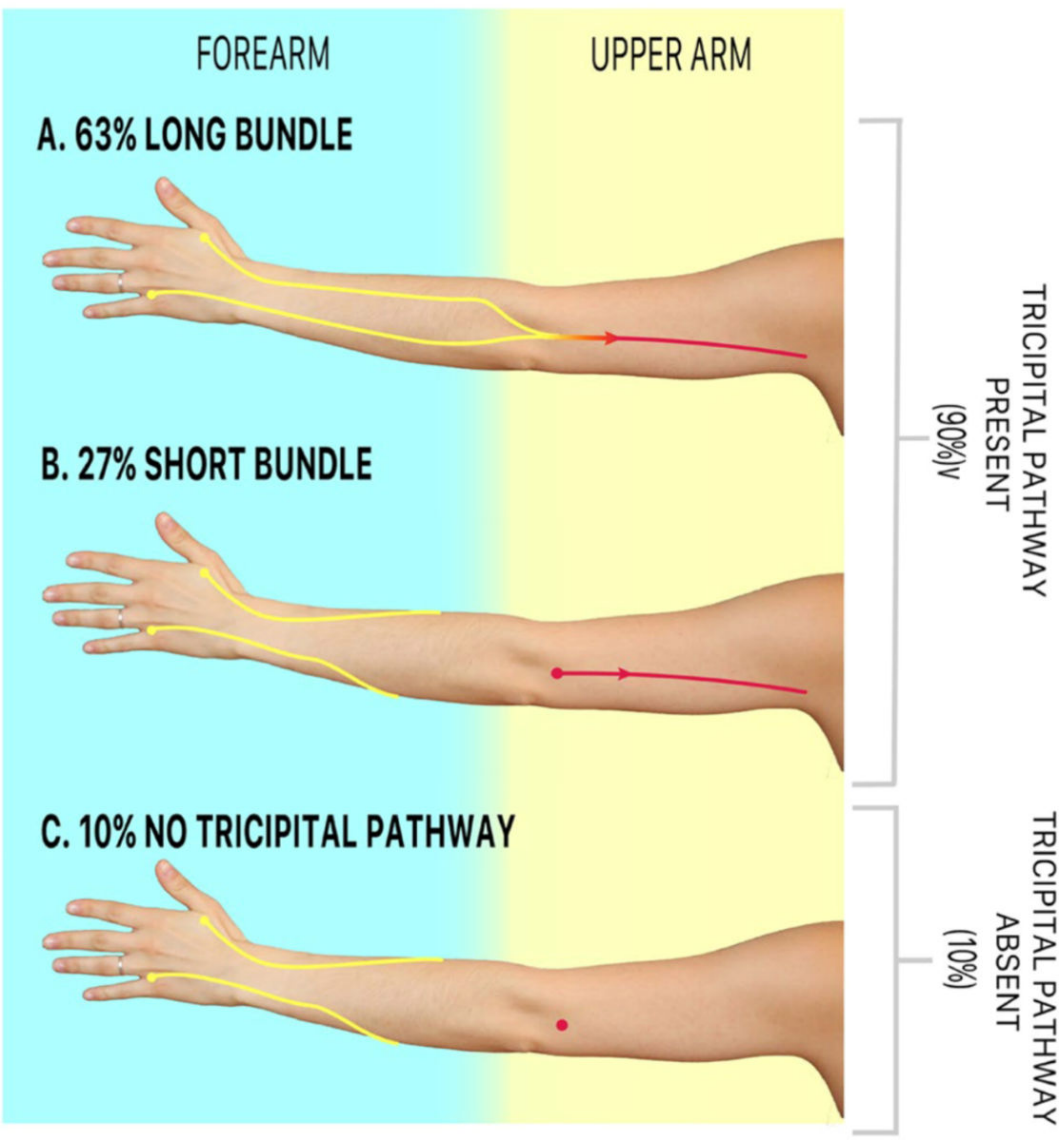
Long and short bundle phenotypes of the tricipital lymphatic channel (reused with permission, Friedman *et al.* 2022, Breast Cancer Research and Treatment^[99]^).

Though we have observed significant variation in baseline lymphatic anatomy between individuals, we have noticed distinct trends in both the main channels and compensatory lymphatic channels [Figure 4]. In 102 preoperative ICG lymphographies performed, we observed that the main pathways arising from the hand and forearm (posterior radial, posterior ulnar, anterior radial, and anterior ulnar) often demonstrate a functional connection to one of two channels in the upper arm: the medial and lateral upper arm channels^[100]^. We also noticed variations in the connectivity of the lateral upper arm channel to the forearm channels, specifically long and short bundle phenotypes [Figure 5]^[93]^. The long bundle lateral upper arm channel is defined as having a functional connection with a forearm channel, most commonly, the posterior radial channel. In the short bundle phenotype, the lateral upper arm channel lacks a functional connection to the forearm channels and is only visualized following the targeted injection over the cephalic vein. Upon postoperative surveillance of 60 patients who underwent ALND, the short bundle lateral upper arm pathway appeared to act as an anatomic risk factor for BCRL^[101]^. We hypothesize that these findings were due to the short bundle phenotype resulting in a watershed region of lymphatic drainage between the forearm and upper arm. We have also observed analogous anatomic phenotypes in the tricipital pathway [Figure 6]. We believe that future investigations focusing on the anatomical variability of this and other compensatory channels such as the tricipital pathway, will help patients at the greatest risk for BCRL development^[99]^.

## FUTURE DIRECTIONS

This knowledge can be applied clinically at various levels of care in both the preoperative and postoperative settings. For the lymphatic surgeon, this information may inform which patients would benefit most from the ILR procedure. Ideally, every patient undergoing ALND would have access to ILR for the prevention of lymphedema despite their anatomical phenotype, as the morbidity of the procedure is quite low. However, the relative inaccessibility to lymphatic surgery and inconsistent healthcare coverage for ILR hinders patients’ ability to access and undergo ILR. Preoperative mapping of lymphatic anatomy using ICG lymphography can be accomplished in an outpatient clinical setting and does not require a lymphatic surgeon. Therefore, this is a feasible way to identify patients at the greatest risk for lymphedema development and for whom ILR would be most beneficial.

Moreover, a better understanding of lymphatic anatomy may inform which lymphatic channels should be prioritized for bypass or identified with an additional dye, the channels in closer proximity to the axillary vein. This knowledge would be important not only to the lymphatic surgeon, but also to members of the tumor board. For example, oncologists may choose to consider anatomical risk when determining a patient’s neoadjuvant chemotherapy regimen and avoid taxane-based regimens altogether when possible. Postoperatively, patients with high-risk anatomy can follow a more rigorous lymphedema surveillance protocol or wear compression garments prophylactically^[102]^. Additionally, understanding compensatory lymphatic channels can help guide both physical therapists and patients in performing manual lymphatic drainage^[103]^. Finally, anatomical knowledge can possibly inform radiotherapy planning and field design in efforts to protect collateral drainage pathways from radiation exposure^[104]^.

Finally, non-surgical methods for the prevention of lymphedema continue to be investigated. The use of pharmaceuticals that promote lymphangiogensis has been developed as potential treatment for lymphedema^[105]^. These drugs could potentially be applied to lymphedema prevention by enabling collateral growth of lymphatic vessels, thereby allowing for continued lymphatic flow after ALND. Further investigation into methods of pharmacological treatment and prevention for lymphedema via lymphangiogenic cytokine delivery, anti-inflammatory agents, as well as anti-fibrotic agents could aid in the non-surgical prevention and treatment of BCRL^[105]^.

## CONCLUSION

The development of breast cancer-related lymphedema following breast cancer treatment is multifactorial and surgical prevention with ILR can reduce the rate of BCRL development^[13]^. Although our understanding of risk factors has evolved, currently established risk factors do not fully account for the variation in BCRL development at the individual level^[106]^. A deeper appreciation of lymphatic anatomy will help to further our understanding of the pathologic changes that occur in BCRL and will help to explain why only a subset of patients develop BCRL after oncologic treatment and ILR. Therefore, there is high utility and value in anatomical mapping of the lymphatic system for both the patient and surgeon.

## Supplementary Material

Video

## Data Availability

Not applicable.

## References

[1] Gillespie TC, Sayegh HE, Brunelle CL, Daniell KM, Taghian AG. Breast cancer-related lymphedema: risk factors, precautionary measures, and treatments. Gland Surg 2018;7:379–403. 30175055 10.21037/gs.2017.11.04 PMC6107585

[2] Armer JM, Radina ME, Porock D, Culbertson SD. Predicting breast cancer-related lymphedema using self-reported symptoms. Nurs Res 2003;52:370–9. 14639083 10.1097/00006199-200311000-00004

[3] Fu MR, Rosedale M. Breast cancer survivors’ experiences of lymphedema-related symptoms. J Pain Symptom Manage 2009;38:849–59. 19819668 10.1016/j.jpainsymman.2009.04.030 PMC2795115

[4] Jørgensen MG, Hermann AP, Madsen AR, Cellulitis is associated with severe breast cancer-related lymphedema: an observational study of tissue composition. Cancers 2021;13:3584. 34298799 10.3390/cancers13143584 PMC8303539

[5] Jørgensen MG, Toyserkani NM, Hansen FG, Bygum A, Sørensen JA. The impact of lymphedema on health-related quality of life up to 10 years after breast cancer treatment. NPJ Breast Cancer 2021;7:70. 34075045 10.1038/s41523-021-00276-y PMC8169644

[6] Khan F, Amatya B, Pallant JF, Rajapaksa I. Factors associated with long-term functional outcomes and psychological sequelae in women after breast cancer. Breast 2012;21:314–20. 22342676 10.1016/j.breast.2012.01.013

[7] Boyages J, Xu Y, Kalfa S, Financial cost of lymphedema borne by women with breast cancer. Psychooncology 2017;26:849–55. 27479170 10.1002/pon.4239 PMC5484300

[8] Johnson AR, Asban A, Granoff MD, Is Immediate lymphatic reconstruction cost-effective? Ann Surg 2021;274:e581–8. 31850991 10.1097/SLA.0000000000003746

[9] Shaitelman SF, Cromwell KD, Rasmussen JC, Recent progress in the treatment and prevention of cancer-related lymphedema. CA Cancer J Clin 2015;65:55–81. 25410402 10.3322/caac.21253 PMC4808814

[10] Martínez-Jaimez P, Armora Verdú M, Forero CG, Breast cancer-related lymphoedema: risk factors and prediction model. J Adv Nurs 2022;78:765–75. 34363640 10.1111/jan.15005

[11] Johnson AR, Kimball S, Epstein S, Lymphedema incidence after axillary lymph node dissection: quantifying the impact of radiation and the lymphatic microsurgical preventive healing approach. Ann Plast Surg 2019;82:S234–41. 30855393 10.1097/SAP.0000000000001864

[12] DiSipio T, Rye S, Newman B, Hayes S. Incidence of unilateral arm lymphoedema after breast cancer: a systematic review and meta-analysis. Lancet Oncol 2013;14:500–15. 23540561 10.1016/S1470-2045(13)70076-7

[13] Hill WKF, Deban M, Platt A, Rojas-Garcia P, Jost E, Temple-Oberle C. Immediate lymphatic reconstruction during axillary node dissection for breast cancer: a systematic review and meta-analysis. Plast Reconstr Surg Glob Open 2022;10:e4291. 35558135 10.1097/GOX.0000000000004291 PMC9084431

[14] Pereira ACP, Koifman RJ, Bergmann A. Incidence and risk factors of lymphedema after breast cancer treatment: 10 years of follow-up. Breast 2017;36:67–73. 28992556 10.1016/j.breast.2017.09.006

[15] Montagna G, Zhang J, Sevilimedu V, Risk factors and racial and ethnic disparities in patients with breast cancer-related lymphedema. JAMA Oncol 2022;8:1195–200. 35679026 10.1001/jamaoncol.2022.1628 PMC9185510

[16] Ahmad A. Breast Cancer Statistics: Recent Trends. In: Ahmad A, editor. Breast cancer metastasis and drug resistance. Cham: Springer International Publishing; 2019. pp. 1–7.

[17] Schulze H, Nacke M, Gutenbrunner C, Hadamitzky C. Worldwide assessment of healthcare personnel dealing with lymphoedema. Health Econ Rev 2018;8:10. 29663122 10.1186/s13561-018-0194-6 PMC5901432

[18] Duhon BH, Phan TT, Taylor SL, Crescenzi RL, Rutkowski JM. Current mechanistic understandings of lymphedema and lipedema: tales of fluid, fat, and fibrosis. Int J Mol Sci 2022;23:6621. 35743063 10.3390/ijms23126621 PMC9223758

[19] Avraham T, Zampell JC, Yan A, Th2 differentiation is necessary for soft tissue fibrosis and lymphatic dysfunction resulting from lymphedema. FASEB J 2013;27:1114–26. 23193171 10.1096/fj.12-222695 PMC3574290

[20] Savetsky IL, Ghanta S, Gardenier JC, Th2 cytokines inhibit lymphangiogenesis. PLoS One 2015;10:e0126908. 26039103 10.1371/journal.pone.0126908 PMC4454507

[21] Ogata F, Fujiu K, Matsumoto S, Excess lymphangiogenesis cooperatively induced by macrophages and CD4(+) T cells drives the pathogenesis of lymphedema. J Invest Dermatol 2016;136:706–14. 27015456 10.1016/j.jid.2015.12.001

[22] Baik JE, Park HJ, Kataru RP, TGF-β1 mediates pathologic changes of secondary lymphedema by promoting fibrosis and inflammation. Clin Transl Med 2022;12:e758. 35652284 10.1002/ctm2.758 PMC9160979

[23] Hespe G, Nitti M, Mehrara B. Pathophysiology of lymphedema. Available from:10.1002/jso.24414 27566412

[24] Aschen S, Zampell JC, Elhadad S, Weitman E, De Brot Andrade M, Mehrara BJ. Regulation of adipogenesis by lymphatic fluid stasis: part II. expression of adipose differentiation genes. Plast Reconstr Surg 2012;129:838–47. 22456356 10.1097/PRS.0b013e3182450b47 PMC3445411

[25] Miaskowski C, Dodd M, Paul SM, Lymphatic and angiogenic candidate genes predict the development of secondary lymphedema following breast cancer surgery. PLoS One 2013;8:e60164. 23613720 10.1371/journal.pone.0060164 PMC3629060

[26] Finegold DN, Baty CJ, Knickelbein KZ, Connexin 47 mutations increase risk for secondary lymphedema following breast cancer treatment. Clin Cancer Res 2012;18:2382–90. 22351697 10.1158/1078-0432.CCR-11-2303 PMC3625665

[27] Finegold DN, Schacht V, Kimak MA, HGF and MET mutations in primary and secondary lymphedema. Lymphat Res Biol 2008;6:65–8. 18564920 10.1089/lrb.2008.1524 PMC4298750

[28] Rockson SG, Keeley V, Kilbreath S, Szuba A, Towers A. Cancer-associated secondary lymphoedema. Nat Rev Dis Primers 2019;5:22. 30923312 10.1038/s41572-019-0072-5

[29] Sudduth CL, Greene AK. Primary lymphedema: update on genetic basis and management. Adv Wound Care 2022;11:374–81. 10.1089/wound.2020.1338 PMC9051871 33502936

[30] Ahmed RL, Schmitz KH, Prizment AE, Folsom AR. Risk factors for lymphedema in breast cancer survivors, the iowa women’s health study. Breast Cancer Res Treat 2011;130:981–91. 21761159 10.1007/s10549-011-1667-z PMC4091732

[31] Tsai RJ, Dennis LK, Lynch CF, Snetselaar LG, Zamba GK, Scott-Conner C. The risk of developing arm lymphedema among breast cancer survivors: a meta-analysis of treatment factors. Ann Surg Oncol 2009;16:1959–72. 19365624 10.1245/s10434-009-0452-2

[32] Yusof KM, Avery-Kiejda KA, Ahmad Suhaimi S, Assessment of potential risk factors and skin ultrasound presentation associated with breast cancer-related lymphedema in long-term breast cancer survivors. Diagnostics 2021;11:1303. 34441238 10.3390/diagnostics11081303 PMC8393908

[33] Armer JM, Ballman KV, McCall L, Factors associated with lymphedema in women with node-positive breast cancer treated with neoadjuvant chemotherapy and axillary dissection. JAMA Surg 2019;154:800–9. 31314062 10.1001/jamasurg.2019.1742 PMC6647005

[34] Ariel IM, Resnick MI, Oropeza R. The effects of irradiation (external and internal) on lymphatic dynamics. Am J Roentgenol Radium Ther Nucl Med 1967;99:404–14. 10.2214/ajr.99.2.404 4960126

[35] LENZI M, BASSANI G. The effect of radiation on the lymph and on the lymph vessels. Radiology 1963;80:814–7. 13929675 10.1148/80.5.814

[36] Allam O, Park KE, Chandler L, The impact of radiation on lymphedema: a review of the literature. Gland Surg 2020;9:596–602. 32420295 10.21037/gs.2020.03.20 PMC7225495

[37] Warren LE, Miller CL, Horick N, The impact of radiation therapy on the risk of lymphedema after treatment for breast cancer: a prospective cohort study. Int J Radiat Oncol Biol Phys 2014;88:565–71. 24411624 10.1016/j.ijrobp.2013.11.232 PMC3928974

[38] Manirakiza A, Irakoze L, Shui L, Manirakiza S, Ngendahayo L. Lymphoedema after breast cancer treatment is associated with higher body mass index: a systematic review and meta-analysis. East Afr Health Res J 2019;3:178–92. 34308212 10.24248/EAHRJ-D-19-00009 PMC8279288

[39] Wu R, Huang X, Dong X, Zhang H, Zhuang L. Obese patients have higher risk of breast cancer-related lymphedema than overweight patients after breast cancer: a meta-analysis. Ann Transl Med 2019;7:172. 31168453 10.21037/atm.2019.03.44 PMC6526274

[40] Ridner SH, Dietrich MS, Stewart BR, Armer JM. Body mass index and breast cancer treatment-related lymphedema. Support Care Cancer 2011;19:853–7. 21240649 10.1007/s00520-011-1089-9 PMC4480912

[41] Mehrara BJ, Greene AK. Lymphedema and obesity: is there a link? Plast Reconstr Surg 2014;134:154e–60e. 10.1097/PRS.0000000000000268 PMC4393748 25028830

[42] Zhu W, Li D, Li X, Association between adjuvant docetaxel-based chemotherapy and breast cancer-related lymphedema. Anticancer Drugs 2017;28:350–5. 27997437 10.1097/CAD.0000000000000468

[43] Hugenholtz-Wamsteker W, Robbeson C, Nijs J, Hoelen W, Meeus M. The effect of docetaxel on developing oedema in patients with breast cancer: a systematic review. Eur J Cancer Care 2016;25:269–79. 10.1111/ecc.12261 25348689

[44] Kim M, Kim SW, Lee SU, A model to estimate the risk of breast cancer-related lymphedema: combinations of treatment-related factors of the number of dissected axillary nodes, adjuvant chemotherapy, and radiation therapy. Int J Radiat Oncol Biol Phys 2013;86:498–503. 23541809 10.1016/j.ijrobp.2013.02.018

[45] Cariati M, Bains SK, Grootendorst MR, Adjuvant taxanes and the development of breast cancer-related arm lymphoedema. Br J Surg 2015;102:1071–8. 26040263 10.1002/bjs.9846

[46] Kilbreath SL, Refshauge KM, Beith JM, Risk factors for lymphoedema in women with breast cancer: a large prospective cohort. Breast 2016;28:29–36. 27183497 10.1016/j.breast.2016.04.011

[47] Swaroop MN, Ferguson CM, Horick NK, Impact of adjuvant taxane-based chemotherapy on development of breast cancer-related lymphedema: results from a large prospective cohort. Breast Cancer Res Treat 2015;151:393–403. 25940996 10.1007/s10549-015-3408-1 PMC4432026

[48] Johnson AR, Granoff MD, Lee BT, Padera TP, Bouta EM, Singhal D. The impact of taxane-based chemotherapy on the lymphatic system. Ann Plast Surg 2019;82:S173–8. 30855384 10.1097/SAP.0000000000001884

[49] Specht MC, Miller CL, Skolny MN, Residual lymph node disease after neoadjuvant chemotherapy predicts an increased risk of lymphedema in node-positive breast cancer patients. Ann Surg Oncol 2013;20:2835–41. 23689935 10.1245/s10434-012-2828-y

[50] Jung SY, Shin KH, Kim M, Treatment factors affecting breast cancer-related lymphedema after systemic chemotherapy and radiotherapy in stage II/III breast cancer patients. Breast Cancer Res Treat 2014;148:91–8. 25253173 10.1007/s10549-014-3137-x

[51] Wilke LG, McCall LM, Posther KE, Surgical complications associated with sentinel lymph node biopsy: results from a prospective international cooperative group trial. Ann Surg Oncol 2006;13:491–500. 16514477 10.1245/ASO.2006.05.013

[52] Kim M, Park IH, Lee KS, Breast cancer-related lymphedema after neoadjuvant chemotherapy. Cancer Res Treat 2015;47:416–23. 25544575 10.4143/crt.2014.079 PMC4506114

[53] Hayes SC, Janda M, Cornish B, Battistutta D, Newman B. Lymphedema after breast cancer: incidence, risk factors, and effect on upper body function. J Clin Oncol 2008;26:3536–42. 18640935 10.1200/JCO.2007.14.4899

[54] Shang T, Liang J, Kapron CM, Liu J. Pathophysiology of aged lymphatic vessels. Aging 2019;11:6602–13. 31461408 10.18632/aging.102213 PMC6738433

[55] Kwan ML, Yao S, Lee VS, Race/ethnicity, genetic ancestry, and breast cancer-related lymphedema in the pathways study. Breast Cancer Res Treat 2016;159:119–29. 27449493 10.1007/s10549-016-3913-x PMC5010992

[56] Meeske KA, Sullivan-Halley J, Smith AW, Risk factors for arm lymphedema following breast cancer diagnosis in Black women and White women. Breast Cancer Res Treat 2009;113:383–91. 18297429 10.1007/s10549-008-9940-5

[57] Guliyeva G, Huayllani MT, Boczar D, Avila FR, Lu X, Forte AJ. Age as a risk factor for breast cancer-related lymphedema: a systematic review. J Cancer Surviv 2023;17:246–53. 33486706 10.1007/s11764-021-00994-z

[58] Zou L, Liu FH, Shen PP, The incidence and risk factors of related lymphedema for breast cancer survivors post-operation: a 2-year follow-up prospective cohort study. Breast Cancer 2018;25:309–14. 29397555 10.1007/s12282-018-0830-3

[59] Byun HK, Kim JS, Chang JS, Validation of a nomogram for predicting the risk of lymphedema following contemporary treatment for breast cancer: a large multi-institutional study (KROG 20–05). Breast Cancer Res Treat 2022;192:553–61. 35107713 10.1007/s10549-021-06507-x

[60] Siotos C, Hassanein AH, Bello RJ, Delayed breast reconstruction on patients with upper extremity lymphedema: a systematic review of the literature and pooled analysis. Ann Plast Surg 2018;81:730–5. 29944525 10.1097/SAP.0000000000001542

[61] Basta MN, Fischer JP, Kanchwala SK, A propensity-matched analysis of the influence of breast reconstruction on subsequent development of lymphedema. Plast Reconstr Surg 2015;136:134e–43e. 10.1097/PRS.0000000000001417 26218386

[62] McLaughlin SA, Brunelle CL, Taghian A. Breast Cancer-related lymphedema: risk factors, screening, management, and the impact of locoregional treatment. J Clin Oncol 2020;38:2341–50. 32442064 10.1200/JCO.19.02896 PMC7343436

[63] Yamada Y. The studies on lymphatic venous anastomosis in lvmphedema. Available from: https://scholar.google.com/scholar?hl=zh-CN&as_sdt=0%2C5&q=Yamada+Y.+The+Studies+on+Lymphatic+Venous+Anastomosis+in+Lvmphedema.+Nagoya+J+Med+Sci.+1969%3B32%3A1-21.&btnG [Last accessed on 22 May 2023]

[64] Boccardo F, Casabona F, De Cian F, Lymphedema microsurgical preventive healing approach: a new technique for primary prevention of arm lymphedema after mastectomy. Ann Surg Oncol 2009;16:703–8. 19139964 10.1245/s10434-008-0270-y

[65] Boccardo F, Casabona F, De Cian F, Lymphatic microsurgical preventing healing approach (LYMPHA) for primary surgical prevention of breast cancer-related lymphedema: over 4 years follow-up. Microsurgery 2014;34:421–4. 24677148 10.1002/micr.22254

[66] Johnson AR, Singhal D. Immediate lymphatic reconstruction. J Surg Oncol 2018;118:750–7. 30114329 10.1002/jso.25177

[67] Coriddi M, Mehrara B, Skoracki R, Singhal D, Dayan JH. Immediate lymphatic reconstruction: technical points and literature review. Plast Reconstr Surg Glob Open 2021;9:e3431. 33680675 10.1097/GOX.0000000000003431 PMC7929616

[68] Wiser I, Weinstein A, Kenworthy E, Mehrara B, Dayan J. Standardizing upper extremity indocyanine green lymphography in a lymphedema outpatient setting. Plast Reconstr Surg Glob Open 2020;8:45.

[69] Spiguel L, Shaw C, Katz A, Fluorescein isothiocyanate: a novel application for lymphatic surgery. Ann Plast Surg 2017;78:S296–8. 28328630 10.1097/SAP.0000000000001034

[70] Russell PS, Velivolu R, Maldonado Zimbrón VE, Fluorescent tracers for in vivo imaging of lymphatic targets. Front Pharmacol 2022;13:952581. 35935839 10.3389/fphar.2022.952581 PMC9355481

[71] Abdallah M, Müllertz OO, Styles IK, Lymphatic targeting by albumin-hitchhiking: Applications and optimisation. J Control Release 2020;327:117–28. 32771478 10.1016/j.jconrel.2020.07.046

[72] Trevaskis NL, Kaminskas LM, Porter CJ. From sewer to saviour - targeting the lymphatic system to promote drug exposure and activity. Nat Rev Drug Discov 2015;14:781–803. 26471369 10.1038/nrd4608

[73] Mele A, Fan B, Pardo J, Axillary lymph node dissection in the era of immediate lymphatic reconstruction: Considerations for the breast surgeon. J Surg Oncol 2021;123:842–5. 33524160 10.1002/jso.26355

[74] Visconti G, Yamamoto T, Hayashi N, Hayashi A. Ultrasound-assisted lymphaticovenular anastomosis for the treatment of peripheral lymphedema. Plast Reconstr Surg 2017;139:1380e–1e. 10.1097/PRS.0000000000003362 28406824

[75] Caretto AA, Stefanizzi G, Garganese G, Treatment of early-stage gynecological cancer-related lower limb lymphedema by lymphaticovenular anastomosis-the triple incision approach. Medicina 2022;58:631. 35630048 10.3390/medicina58050631 PMC9143574

[76] Schwarz GS, Grobmyer SR, Djohan RS, Axillary reverse mapping and lymphaticovenous bypass: Lymphedema prevention through enhanced lymphatic visualization and restoration of flow. J Surg Oncol 2019;120:160–7. 31144329 10.1002/jso.25513

[77] Gagne P, Sharma K. Relationship of common vascular anatomy to cannulated catheters. Int J Vasc Med 2017;2017:5157914. 10.1155/2017/5157914 PMC5749287 29410917

[78] Nomoto S, Hirakawa K, Ogawa R. Safety of copolyamide filler injection for breast augmentation. Plast Reconstr Surg Glob Open 2021;9:e3296. 33680632 10.1097/GOX.0000000000003296 PMC7929552

[79] Yamamoto T, Yamamoto N, Yamashita M, Furuya M, Hayashi A, Koshima I. Efferent lymphatic vessel anastomosis: supermicrosurgical efferent lymphatic vessel-to-venous anastomosis for the prophylactic treatment of subclinical lymphedema. Ann Plast Surg 2016;76:424–7. 25389716 10.1097/SAP.0000000000000381

[80] Gentileschi S, Albanese R, Pino V, SPECT/CT and fusion ultrasound to target the efferent groin lymph node for lymphatic surgery. Microsurgery 2019;39:605–12. 31400162 10.1002/micr.30501

[81] Gentileschi S, Servillo M, Garganese G, The lymphatic superficial circumflex iliac vessels deep branch perforator flap: a new preventive approach to lower limb lymphedema after groin dissection-preliminary evidence. Microsurgery 2017;37:564–73. 27987230 10.1002/micr.30142

[82] Caretto AA, Stefanizzi G, Fragomeni SM, Lymphatic function of the lower limb after groin dissection for vulvar cancer and reconstruction with lymphatic SCIP flap. Cancers 2022;14:1076. 35205824 10.3390/cancers14041076 PMC8870617

[83] Samra F, Sobti N, Nelson JA, Allen RJ Jr, Mehrara B, Dayan JH. Frontiers in oncologic reconstruction. Plast Reconstr Surg Glob Open 2019;7:e2181. 31624664 10.1097/GOX.0000000000002181 PMC6635183

[84] Chen WF, Knackstedt R. Delayed distally based prophylactic lymphaticovenular anastomosis: improved functionality, feasibility, and oncologic safety? J Reconstr Microsurg 2020;36:e1–2. 10.1055/s-0040-1716743 32957152

[85] Johnson AR, Otenti D, Bates K, Creating a policy for coverage of lymphatic surgery: addressing a critical unmet need. Plast Reconstr Surg 2023; 10.1097/prs.0000000000010239. 36727781

[86] Johnson AR, Fleishman A, Tran BNN, Developing a lymphatic surgery program: a first-year review. Plast Reconstr Surg 2019;144:975e–85e. 10.1097/PRS.0000000000006223 31764631

[87] Bracaglia R, D’Ettorre M, Gentileschi S, Tambasco D. Was the surgeon a satisfactory informant? Aesthet Surg J 2014;34:632–5. 24755417 10.1177/1090820X14528504

[88] Sappey M, Karmanski A, Beau E, Bry A. Anatomie, Physiologie, Pathologie de Vaisseaux Lymphatiques. Adrain Delahaye, Imprimerie de E. Martinet; 1874. Available from: https://archive.org/details/BIUSante_01562/page/n1/mode/2up [Last accessed on 22 May 2023]

[89] Suami H. Lymphosome concept: Anatomical study of the lymphatic system. J Surg Oncol 2017;115:13–7. 27334241 10.1002/jso.24332

[90] Suami H, Scaglioni MF. Anatomy of the lymphatic system and the lymphosome concept with reference to lymphedema. Semin Plast Surg 2018;32:5–11. 29636647 10.1055/s-0038-1635118 PMC5891651

[91] Tourani SS, Taylor GI, Ashton MW. Anatomy of the superficial lymphatics of the abdominal wall and the upper thigh and its implications in lymphatic microsurgery. J Plast Reconstr Aesthet Surg 2013;66:1390–5. 23746863 10.1016/j.bjps.2013.05.030

[92] Johnson AR, Bravo MG, James TA, Suami H, Lee BT, Singhal D. The all but forgotten mascagni-sappey pathway: learning from immediate lymphatic reconstruction. J Reconstr Microsurg 2020;36:28–31. 31398762 10.1055/s-0039-1694757

[93] Kubik S. The role of the lateral upper arm bundle and the lymphatic watersheds in the formation of collateral pathways in lymphedema. Acta bio Acad Sci Hung 1980;31:191–200. 7223234

[94] Johnson AR, Granoff MD, Suami H, Lee BT, Singhal D. Real-time visualization of the mascagni-sappey pathway utilizing ICG lymphography. Cancers 2020;12:1195. 32397246 10.3390/cancers12051195 PMC7281680

[95] Ciucci J. Derivative lymphatic currents UL, Ciucci, JL - English. In: Linfedema Del Miembro Superior Postratamiento Del Cancer de Mama. Nayarit Ed; 2004:29. Available from: https://linfedemahoy.com/linfedema-de-miembro-superior [Last accessed on 22 May 2023]

[96] Latorre J, Ciucci JL, Rosendo A, Anatomía Del Sistema Linfático Del Miembro Superior Revisión. Available from: https://filadd.com [Last accessed on 22 May 2023]

[97] Ciucci JL, Vadra GD, Sorocco J. Investigación anatómica del drenaje linfático del miembro superior. Su importancia en la patología traumatológica. Available from: https://www.aaot.org.ar/revista/1993_2002/1997/1997_4/620413.pdf [Last accessed on 22 May 2023]

[98] Amore M, Tapia L, Mercado D, Pattarone G, Ciucci J. Lymphedema: a general outline of its anatomical base. J Reconstr Microsurg 2016;32:2–9. 26375305 10.1055/s-0035-1560038

[99] Friedman R, Bustos VP, Pardo J, Superficial and functional imaging of the tricipital lymphatic pathway: a modern reintroduction. Breast Cancer Res Treat 2023;197:235–42. 36326995 10.1007/s10549-022-06777-z PMC10691657

[100] Granoff MD, Pardo JA, Johnson AR, Superficial and functional lymphatic anatomy of the upper extremity. Plast Reconstr Surg 2022;150:900–7. 35939638 10.1097/PRS.0000000000009555 PMC9674086

[101] Granoff MD, Pardo J, Shillue K, Variable anatomy of the lateral upper arm lymphatic channel: a potential anatomic risk factor for the development of breast cancer related lymphedema. Plast Reconstr Surg 2023. 10.1097/PRS.0000000000010245 36727729

[102] Paramanandam VS, Dylke E, Clark GM, Prophylactic use of compression sleeves reduces the incidence of arm swelling in women at high risk of breast cancer-related lymphedema: a randomized controlled trial. J Clin Oncol 2022;40:2004–12. 35108031 10.1200/JCO.21.02567

[103] Koelmeyer LA, Thompson BM, Mackie H, Personalizing conservative lymphedema management using indocyanine green-guided manual lymphatic drainage. Lymphat Res Biol 2021;19:56–65. 33270517 10.1089/lrb.2020.0090

[104] Gross JP, Sachdev S, Helenowski IB, Radiation therapy field design and lymphedema risk after regional nodal irradiation for breast cancer. Int J Radiat Oncol Biol Phys 2018;102:71–8. 30102206 10.1016/j.ijrobp.2018.03.046

[105] Brown S, Dayan JH, Coriddi M, Pharmacological treatment of secondary lymphedema. Front Pharmacol 2022;13:828513. 10.3389/fphar.2022.828513 PMC8822213 35145417

[106] Shah C, Vicini FA. Breast cancer-related arm lymphedema: incidence rates, diagnostic techniques, optimal management and risk reduction strategies. Int J Radiat Oncol Biol Phys 2011;81:907–14. 21945108 10.1016/j.ijrobp.2011.05.043

